# Growth of bulk β-Ga_2_O_3_ crystals from melt without precious-metal crucible by pulling from a cold container

**DOI:** 10.1038/s41598-024-65420-7

**Published:** 2024-06-27

**Authors:** A. Yoshikawa, V. Kochurikhin, T. Tomida, I. Takahashi, K. Kamada, Y. Shoji, K. Kakimoto

**Affiliations:** 1 Institute for Materials Research, Tohoku University, 2-1-1 Katahira, Sendai, 980-8577 Japan; 2 New Industry Creation Hatchery Center, Tohoku University, 6-6-10 Aoba, Aramaki, Sendai, 980-8579 Japan; 3 C&A Corporation, 1-16-23 Ichibancho, Aoba-ku, Sendai, 980-0811 Japan; 4 Research Institute for Applied Mechanics, Kyushu University, 6-1 Kasuga-koen, Kasuga, Fukuoka, 816-8580 Japan

**Keywords:** Gallium oxide, Oxide crystal growth without precious metal crucible, Melt growth, Cold crucible, Oxide semiconductor, Wide bandgap semiconductor, Engineering, Materials science

## Abstract

We report the growth of bulk β-Ga_2_O_3_ crystals based on crystal pulling from a melt using a cold container without employing a precious-metal crucible. Our approach, named oxide crystal growth from cold crucible (OCCC), is a fusion between the skull-melting and Czochralski methods. The absence of an expensive precious-metal crucible makes this a cost-effective crystal growth method, which is a critical factor in the semiconductor industry. An original construction 0.4–0.5 MHz SiC MOSFET transistor generator with power up to 35 kW was used to successfully grow bulk β-Ga_2_O_3_ crystals with diameters up to 46 mm. Also, an original diameter control system by generator frequency change was applied. In this preliminary study, the full width at half maximum of the X-ray rocking curve from the obtained β-Ga_2_O_3_ crystals with diameters ≤ 46 mm was comparable to those of β-Ga_2_O_3_ produced by edge-defined film fed growth. Moreover, as expected, the purity of the obtained crystals was high because only raw material-derived impurities were detected, and contamination from the process, such as insulation and noble metals, was below the detection limit. Our results indicate that the OCCC technique can be used to produce high-purity bulk β-Ga_2_O_3_ single crystalline substrate.

## Introduction

Compared with traditional wide bandgap materials, such as SiC or GaN, β-Ga_2_O_3_ is expected to help realize energy-saving power devices with higher performance owing to its wider bandgap (4.5–4.9 eV)^1–3^. The development of β-Ga_2_O_3_-based Schottky barrier diodes^1–4^ and field-effect transistors^5–8^ for power electronics is progressing rapidly. Similar to Si, β-Ga_2_O_3_ can be grown from a melt; therefore, β-Ga_2_O_3_ is expected to have comparable low-cost and high-quality characteristics. In addition, owing to the rapid development of Ga_2_O_3_ crystal growth technology, such crystals can find a vast range of applications in optoelectronic devices, including waveguides, photodetectors, and scintillators^9^. However, at present, the most widely used growth methods for bulk oxide single crystals require the use of precious-metal crucibles^10–13^. Growing high-melting-point-oxide crystals requires expensive iridium crucibles, which leads to high production costs for single-crystal substrates. Technoeconomic modeling results published in 2019^14^ showed that the cost of iridium (used as the crucible material) is the major factor determining the total cost of Ga_2_O_3_ wafer. Since the release of this analysis, the price of iridium has increased by more than 3 times. Therefore, Reese et al.^14^ suggest that technological innovations—such as the development of alternative crucible materials that can significantly lower the cost of gallium oxide substrates—can encourage the widespread use of gallium oxide semiconductor devices. The production efficiency can be improved further via crucible-free methods and technological developments to obtain large and high-quality crystals.

In an effort to overcome the problem of decomposition and volatilization in low-oxygen partial-pressure atmospheres, Villora et al.^15^ demonstrated a one-inch diameter and near two-inch-long β-Ga_2_O_3_ crystal grown by the crucible-free Floating Zone (FZ) method in 50% oxygen atmosphere. Hoshikawa et al^16^*.* contributed to the development of a technology for the growth of β-Ga_2_O_3_ bulk single crystals using the Bridgman method with platinum-based alloys. The use of Pt–Rh alloy as a crucible material is an alternative solution that avoids using Ir crucibles and its losses through oxidation. To the grow of a 2-inch boule in air atmosphere has been reported^17^. However, the use of a Pt–Rh crucible caused strong crystal coloration due to Rh impurities in the melt, which leads to limitations in optical applications.

The growth of bulk β-Ga_2_O_3_ crystals at least up to 2 inch in diameter using the Czochralski (CZ) technique was realized. This method requires the use of high oxygen partial pressure and Ir. This issue has been resolved using an exceedingly wide oxygen pressure variation during the different stages of heating and growth^18^. The edge-defined film fed growth (EFG) and CZ methods demonstrated large crystal volumes roughly between 100 and 200 cm^3^ of high structural quality crystals, making them far more economically efficient than any crucible-free methods demonstrated so far. Currently, 4-inch substrates of β-Ga_2_O_3_ grown by the EFG technique are commercially available. However, both techniques require the use of iridium crucibles, which requires a compromise between the oxygen concentration needed for the prevention of β-Ga_2_O_3_ melt decomposition and the strong Ir crucible oxidation at high oxygen concentration.

Recently, we successfully grew Gd–Al–Ga crystals (melting point ~ 1820 °C) using the oxide crystal growth from cold crucible (OCCC) method and avoiding the use of a precious-metal crucible^19^. The OCCC method combines two conventional and well-known methods: the cold crucible method [^20–24^] for the heat source and the CZ method for crystal growth. As the growth of larger diameter crystals requires much higher O_2_ concentration^18^, the significance of developing the OCCC method lies here.

Because this method does not involve Ir oxidation, there are no restrictions on the atmosphere during growth. The melt can be kept at any oxygen concentration in the growth atmosphere, which is expected to significantly reduce the number of oxygen defects in the grown crystals. Recently, a similar technique was successfully applied for the growth of BGO and SrTiO_3_ single crystals using a high-frequency (4–6 MHz) vacuum tube generator as a power source [^25,26^]. In this work, bulk β-Ga_2_O_3_ crystals were grown by the OCCC technique using both a traditional vacuum tube generator and an original construction SiC transistor generator as the power source. To the best of our knowledge, this is the first study that reports the application of this single-crystal growth method to β-Ga_2_O_3_. The proposed method has the potential to serve as an alternative to currently used methods once the technology for the stable production of large β-Ga_2_O_3_ crystals via the OCCC technique is developed.

## Experimental procedure

**Figure 1:**
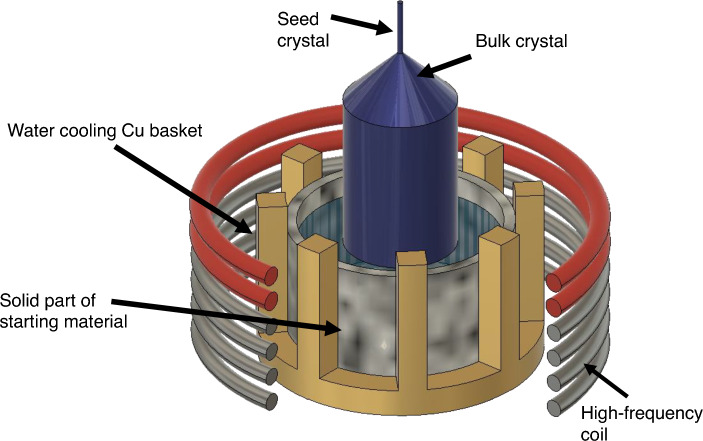
Schematic of the experimental setup, showing the high-frequency coil, water-cooled copper basket, sintered melt retention (cold crucible) area, and melt area.

Figure 1 shows a schematic of the OCCC method. The equipment setup used in this study comprised a high-frequency oscillation coil, a water-cooled Cu basket with cold water flowing inside, a seed pulling and rotation mechanism, and a Y-stabilized Zr ceramic hot zone at the upper part that generated the required temperature distribution on the melt surface. Heating and melt holding were based on the skull-melting method. Briefly, the sintered raw material served as a container to hold the melt. Owing to the low temperature near the Cu basket pipes, a thin layer of the unmelted raw material functioned as a self-container for the melt.

Upon melt stabilization, the growth was similar to that in the conventional CZ method. At room temperature (23 °C), the electric conductivity of pressed Ga_2_O_3_ pellets was insufficient for direct heating by the radio frequency (RF) field. Various materials were used as ignition materials: a small Ir ring removed immediately after the visible melt, 10–20 g of Ga metal evenly distributed in the powder, or a few carbon pieces. In addition, it was observed that after rather rapid melt solidification, the thin area at the periphery of the solidified melt had a black color, probably due to the reduction to metal Ga. This thin layer had sufficient electric conductivity for direct heating without the need for any additional ignition materials.

Brief stages of raw-material charging were not performed. After a first portion of the raw material had been melted, further Ga_2_O_3_ powder was gradually added until the desired melt level in the basket was achieved.

**Figure 2:**
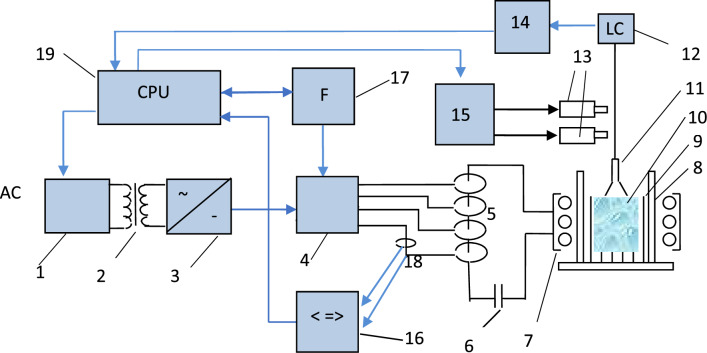
The simplified block diagram of the experimental setup with the use of an original SiC MOSFET transistor generator and diameter control system by the generator frequency control. 1: Thyristor regulator; 2: step-up transformer; 3: rectifier unit; 4: inverter (SiC MOSFET transistor modules bank); 5: set of high frequency toroidal transformers; 6: capacitor assembly; 7: induction coil; 8: water cooled basket (cold container); 9: non-melted layer of powder; 10: melt; 11: growing crystal; 12: weight sensor (load-cell); 13: puling and rotation motors; 14: digital voltmeter; 15: motor controller; 16: power analyzer (Hioki, model PW8001); 17: dual channel Frequency synthesizer (Keysight, 33500B); 18: voltage and current detectors; 19: control computer.

Initially, we made experiments with a traditional power source for the cold crucible technique, with a high frequency of 5 MHz and a 60-kW vacuum tube generator. However, due to the high electric conductivity of the Ga_2_O_3_ melt compared with many other oxide melts, we successfully obtained the full volume of melt at small diameter Cu baskets (˂ 60 mm). For basket sizes of 85–100 mm, unmelted material was observed in the center of the baskets. In our previous research for the crucible-free growth of Gd–Al–Ga crystals, we successfully applied an original 0.4–0.5 MHz SiC MOSFET transistor generator with power up to 35 kW^19^. In this study, the same generator was used for the growth of β-Ga_2_O_3_ crystals with larger size baskets. The block diagram of our experimental setup using a lower frequency SiC power generator is shown in Fig. 2. Despite the limitation on the upper frequency, such a SiC generator is free from the serious disadvantages of a tube generator, such as low efficiency, low vacuum tube lifetime, and high (˂ 15 kV) operational voltage.

β-Ga_2_O_3_ (99.99% purity) powder was used as the starting material. An ordinary air atmosphere was used for the crystal growth at a flow rate in the range of 5–10 L/min. We also experimented performing our experiments under pure oxygen or 100% CO_2_ atmosphere. However, the flow patterns on the melt surface were more irregular and achieving stable crystal growth was difficult.

The dimensions of the main Cu basket were 85 mm × 60 mm. Y-stabilized zirconia was used for the top side insulation. The growth rate was set to 3–5 mm/h, and the rotation speed was set to 4–6 rpm. Thin β-Ga_2_O_3_ crystals grown previously by means of the floating-zone method along the ⟨010⟩ axis were used as the seeds.

Impurities were determined using glow discharge mass spectrometry (GDMS) analysis. X-ray rocking curve (XRC) measurements were performed with a diffraction angle of [400] 2*θ* = 30.1162° using a Ge(220) four-crystal monochromator. The target of the X-ray source was Cu, and the slit dimensions were 1 mm × 1 mm.

## Results and discussion

During our experiments with the Ga_2_O_3_ melting in a cold container, it was found that varying the generator power mainly leads to changes in the melt volume, resulting in only minor changes in the temperature of the melt surface. Moreover, after decreasing the generator power to a certain value, the volume of melt decreased markedly, and due to the power distribution to the remaining melt, its temperature increased further. In the case of the 5 MHz vacuum tube generator (such generators are usually manufactured according to a self-excited circuit), the generator power was the only parameter possible to control. For diameter control, we used a similar method to the one applied before for the growth of Gd-Al-Ga garnet—air flow through a ceramic pipe over the melt surface using a cooling fan placed outside the growth chamber. By varying the speed of the cooling fan, it was possible to change the melt surface temperature and control the growing crystal diameter.

In the experiments with a larger Cu basket and lower frequency SiC generator, we varied the generator frequency across a wide range, in addition to varying the power. This method was found to be more effective for controlling the diameter. The frequency range for the control was 430–470 kHz with constant power generation. During the growth process, periodical fluctuations were observed in the melt temperature similar to that observed in the traditional cold crucible technique. The stabilization of such phenomena was realized by automatic frequency adjustment to maintain a constant phase difference between the voltage and current of high frequency power.

**Figure 3:**
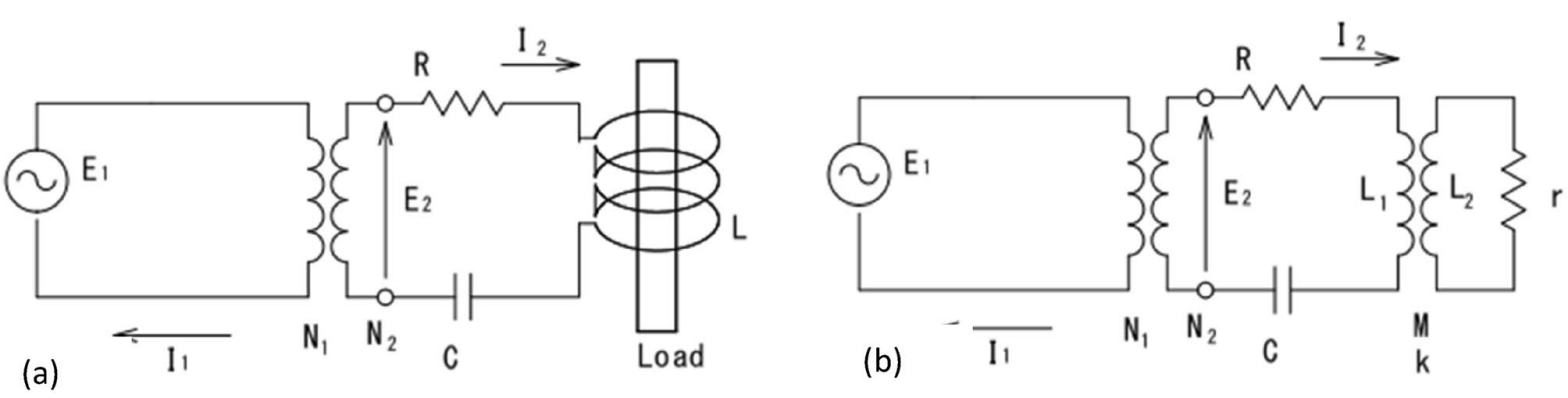
Series resonance circuit (**a**) and circuit with heating object (**b**).

Phase difference control (frequency control) of the oscillator was introduced to observe the progress of melt solidification in the cold container and to stabilize the temperature gradient in the melt by stabilizing the amount of heat generated by the object being heated. In the series resonance circuit shown in Fig. 3a, the phase difference θ is as follows.

$$\theta ={tan}^{-1}{\left(\frac{2\pi fL-\frac{1}{2\pi fC}}{R}\right)}^{2},P=VIcos\theta$$

The equivalent circuit in this figure is shown in Fig. 3b, where r is the resistance of the object to be heated (oxide melt). The resistance R of the circuit is coupled to the object to be heated through the work coil, and when r changes, R also changes according to the coupling coefficient k and mutual inductance M. Therefore, when the volume of the melt changes due to solidification, the resistance of the circuit changes, which can be observed as a phase difference, and controlling the oscillation frequency to keep the phase difference constant prevents a decrease in heat generation and stabilizes the melt state.

**Figure 4:**
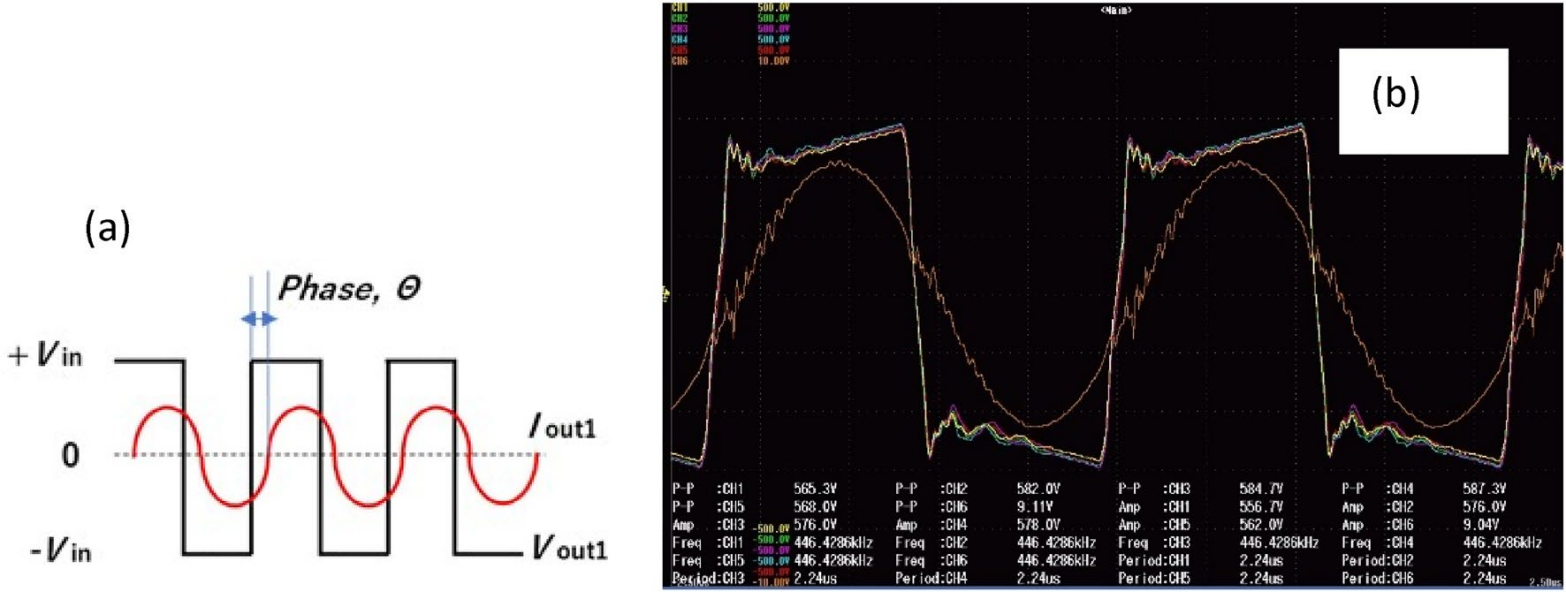
The meaning of phase difference and the real oscilloscope image. Ch. 1–5–output voltage signals of five SiC power modules, Ch. 6 (orange line)–current signal of one SiC module.

In practical experiments, the measurement and comparison of phase signal were made using a digital power analyzer (Pos 16 on Fig. 2). The graphical meaning of phase difference and real image in the oscilloscope at the completely melted Ga_2_O_3_ conditions are shown in Fig. 4. Phase difference close to zero is the most efficient operating mode of the generator. However, to prevent the destruction of SiC transistors in the case of operation at the exact resonance conditions, the automatic system was maintained such that the difference between the magnitudes of the generator and resonance frequencies was at least 1000 Hz.

After the final melting of raw materials, the frequency shift needed for retaining the same phase difference as before the melting was ~ 10–15 kHz. These values were observed with the use of a 4-turn RF coil with a coil height of 50 mm. Our previous experiments on melting Gd-Al-Ga garnet in the same size Cu basket demonstrated that the frequency change after the final melting of the raw materials was ≤ 2–3 kHz^19^. These data are in good agreement with the higher electric conductivity of undoped Ga_2_O_3_ melt. When crystal expansion initiates, the phase difference (and respective melt temperature) gradually increased with increasing generator frequency using the control software.

**Figure 5:**
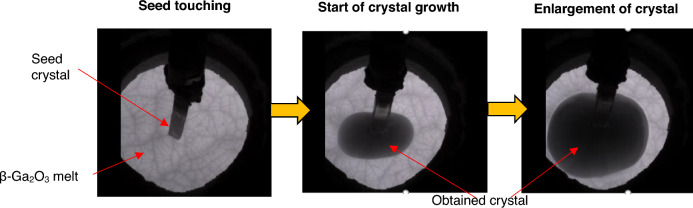
Stages in the OCCC method procedure. From left to right: seed touching, initial crystal growth, and enlargement of the bulk β-Ga_2_O_3_ crystal.

Figure 5 illustrates key steps during the OCCC method processing, from seed touching to the growth of bulk β-Ga_2_O_3_ crystals.

**Figure 6:**
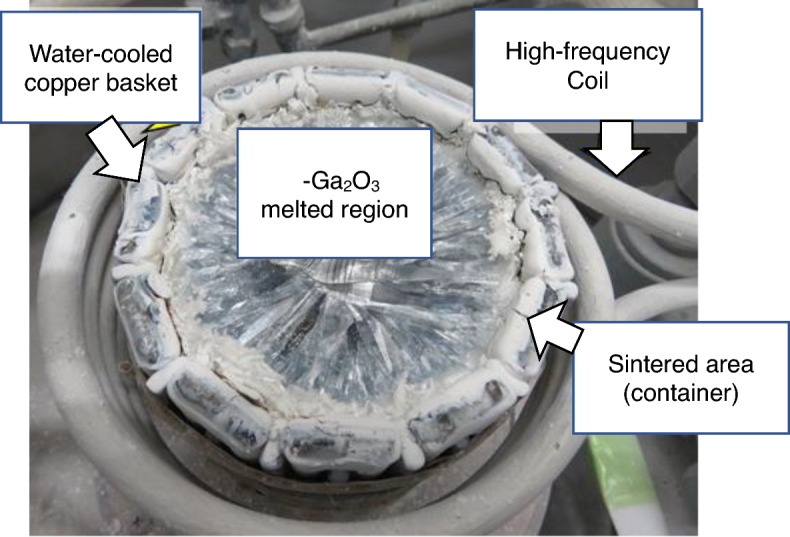
Top view of the copper basket after cooling during β-Ga_2_O_3_ bulk crystal growth via the OCCC method.

Figure 6 shows the internal view of the copper basket after the β-Ga_2_O_3_ bulk crystal growth using the OCCC method is complete. The thin white area lining the copper basket pipes is unmelted sintered Ga_2_O_3_, and an area around the center of the basket contains several transparent crystal blocks that have melted and solidified.

**Figure 7:**
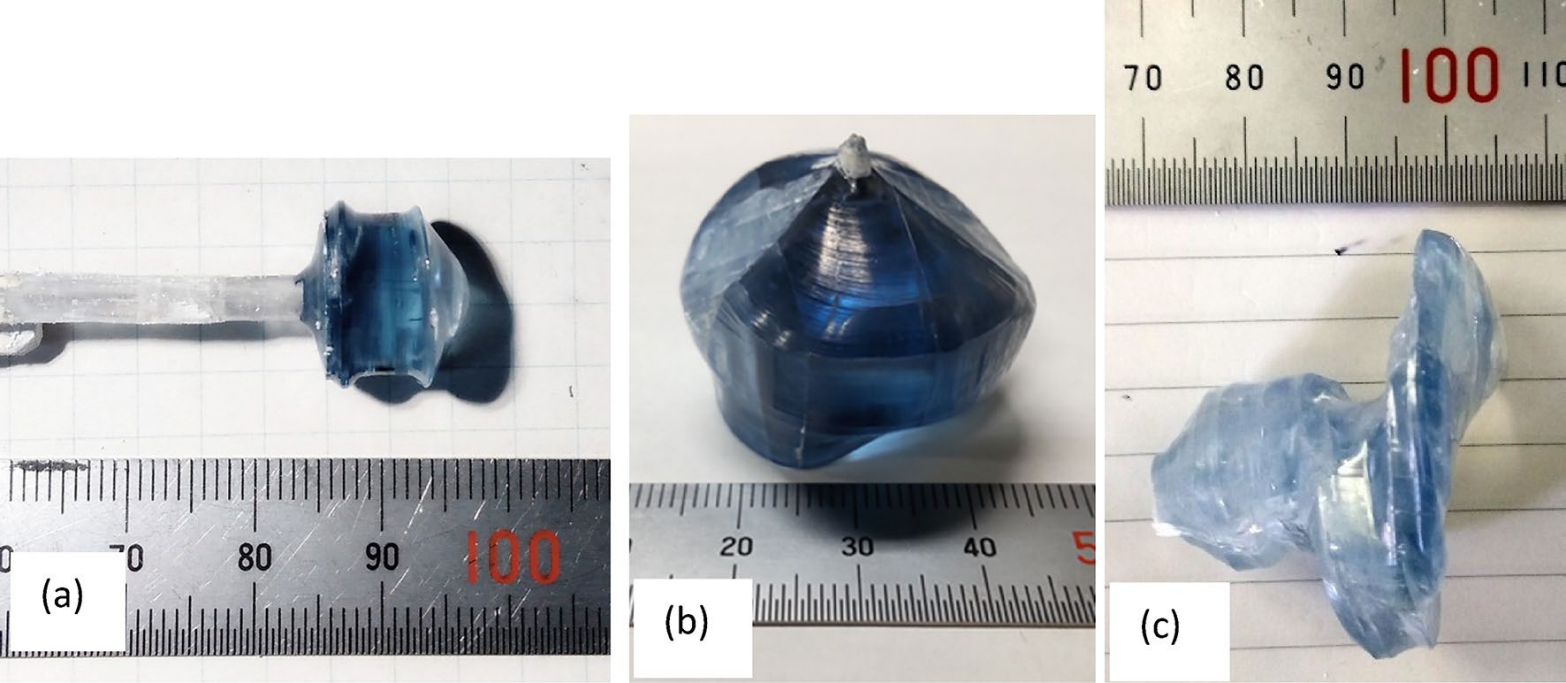
Photographs of β-Ga_2_O_3_ single crystals grown using a 5 MHz generator. (**a**) β-Ga_2_O_3_ single crystal with seed crystal. The size is ~ 10 mm in diameter and ~ 10 mm in body length. (**b**) β-Ga_2_O_3_ single crystal. The size is ~ 1 inch in diameter and ~ 15 mm in body length. (**c**) β-Ga_2_O_3_ single crystal with spiral growth.

Crystals grown using a 60 mm Cu-basket and the 5 MHz generator are shown in Fig. 7. For small (10–15 mm) crystal diameters, the interface shape was slightly convex and diameter control was rather easy. However, upon reaching crystal diameters ~ 20–25 mm, the interface shape became concave and the difficulties in diameter control increased. Often, rapid radial growth to one side of the spiral shape was observed (Fig. 7c). Figure 7b shows the biggest crystal grown using the 60 mm basket and 5 MHz high frequency, which measured 30 mm in diameter and 30 mm in length. Larger diameter crystals were grown using 85- and 100-mm diameter baskets with 430–470 kHz power supply with frequency control.

**Figure 8:**
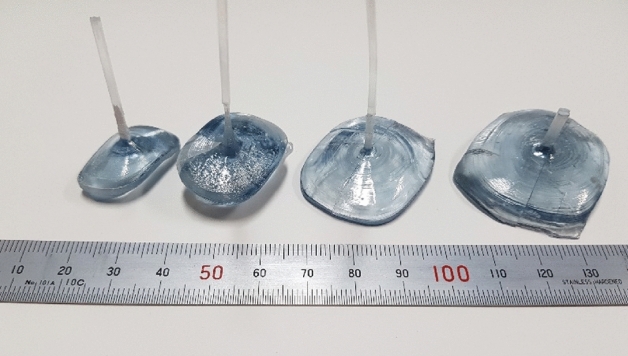
Photograph of β-Ga_2_O_3_ single crystals grown in air using the 430–470 kHz SiC generator.

In our initial experiments, the seed crystals frequently melted during seeding due to unoptimized temperature distribution on the surface of the melt. Careful selection of the operation frequency range along with optimization of the shape of the ceramic hot zone were required for successful seeding. Under the conditions used in the present study, β-Ga_2_O_3_ single crystals with diameter ≤ 46 mm were grown successfully (Fig. 8). All grown crystals were transparent and slightly bluish in color. The solid–liquid interface shape during the growth of β-Ga_2_O_3_ single crystals was strongly dependent on the liquid layer thickness in the 85 mm Cu basket. In the case of relatively short layer thickness (10–15 mm), the interface shape was concave, and stronger melt temperature periodical fluctuations were observed. However, in the melt layer ≥ 20–30 mm, the interface shape was slightly convex and growth was more stable. Melt height within certain limits could be controlled by ignition conditions and the shape of the RF coil. Furthermore, the interface shape may also be controlled via a careful selection of the crystal rotation rate as in conventional CZ growth. The relatively rapid and sharp expansion of the growing crystal observed in several experiments was caused by the steep temperature gradient. In the case of 85–100 mm Cu baskets and lower frequency SiC generator, we also performed the experiments with cold-air flow to the bottom area of the seed holder. This was sufficient to increase the axial gradient and make rapid crystal expansion more controllable. However, it generated rather strong noise to the crystal weight signal (i.e. noise to signal ratio was high) and created additional difficulties for the crystal shape control.

Table 1 presents the results of an impurity evaluation based on GDMS analysis. Si and Fe impurities were observed, whereas the amounts of the materials in the immediate environment of the melt, such as Cu (from the basket) and Zr (from the insulator), were below the detection limit. Si contamination (> 1 ppm), which can be assumed to originate from the raw materials. Fe and other contaminants were believed to have originated from the preparation stage (pressing). The levels of other elements, such as Na, Ti, Mn and Co, Pt, and Ir, were below the detection limit. Pt and Ir were not detected because we did not use a precious-metal crucible; this is a significant difference from the conventional method that uses a precious-metal crucible and impurities of such metals easily can be identified in the grown crystal or solidified melt.

**Table 1:** Results of impurity evaluation based on GDMS analysis (ppm).

	Grown crystal	Powder
Na	0.13	–
Mg	0.04	< 1
Si	11	5
Ca	0.16	< 1
Fe	0.89	< 1
Cu	< 0.05	< 1
Zn	< 0.1	< 1
Ge	< 0.1	< 5
Ti	0.007	–
Mn	< 0.05	–
Co	< 0.01	–
Zr	< 0.05	–
Pt	< 0.05	–
Ir	< 0.01	–

**Figure 9:**
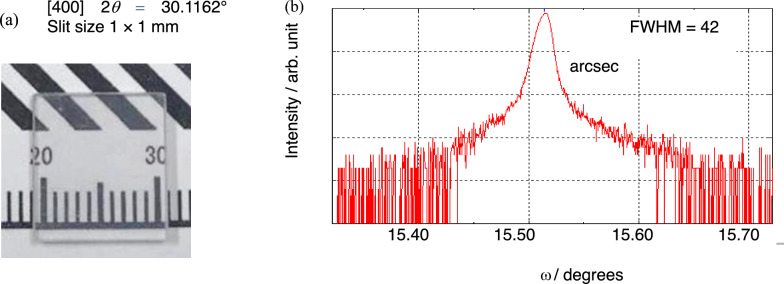
XRC analysis. (**a**) β-Ga_2_O_3_ single crystalline plate used for XRC measurement and (**b**) XRC pattern of the β-Ga_2_O_3_ single crystal grown using the OCCC method.

Figure 9 shows a cut and polished sample of the grown β-Ga_2_O_3_ crystal and the results of the XRC measurement. A full width at half maximum (FWHM) value of 42 arcsec was obtained. A this FWHM value is comparable than that of β-Ga_2_O_3_ grown using the edge-defined film-fed growth method^27^. At present early stage of OCCC method investigation, it is noticeably lower than those of CZ-grown crystals^28^. At the start of crystallization, the grain boundaries were observed mainly at the central part close to the seed. For crystals grown with the convex solid–liquid interface shape, the control of the shape was easier and it was possible to expand the crystal to the desirable diameter without excessively rapid radial growth, which often caused the formation of grain boundaries.

## Conclusions

Bulk β-Ga_2_O_3_ crystals with diameters ≤ 46 mm were successfully grown by pulling from a cold container without using a precious-metal crucible. The OCCC method was used to grow bulk gallium oxide single crystals based on the CZ method, using the cold crucible method to protect the copper basket from the heat source. Gallium oxide single crystals with a maximum diameter of ~ 5 cm were successfully grown. Although this was an initial trial in which neither the penetration depth of the high-frequency magnetic field nor the temperature gradient in the pull-up direction were optimized, the XRC FWHM of the obtained β-Ga_2_O_3_ crystal was comparable to those of β-Ga_2_O_3_ sample grown by the EFG technique. In the impurity measurement, Si from the raw material and Fe, which was probably mixed into the raw material during pelletizing, were found. The Cu content (from the water-cooled Cu basket) and the Zr content (from the Y-stabilized ZrO_2_ insulation material) were below the detection limit. In addition, no Ir impurities were found even when using the Ir ring as ignition material. Although research on crystal growth technology for β-Ga_2_O_3_ via the OCCC method is at an early stage, crystals with a diameter ≥ 30 mm have been successfully grown. Therefore, the barrier to growing large-diameter crystals may not be high. As for longer lengths, an aspect ratio close to 1 could be achieved when a temperature gradient sufficient to suppress spiral growth was established. If an appropriate automatic diameter control system is realized, this method can be used to produce crystals with sufficient wafer size in large quantities. These results indicate that the OCCC crystal growth technique can be used to produce bulk β-Ga_2_O_3_ single crystals, which are increasingly required in the semiconductor and optoelectronics industries owing to the improved application prospects of gallium oxide semiconductors. As cost is an important factor for the wider adaptation of gallium oxide semiconductor devices, the method proposed herein is expected to be an important technology from the perspective of supplying gallium oxide semiconductor substrates at low cost.

## Data Availability

The datasets generated during and/or analyzed during the current study are available from the corresponding author on reasonable request; the data are not publicly available due to a NEDO project cooperative research agreement.
